# The mental health and help-seeking behaviour of resettled Afghan refugees in Australia

**DOI:** 10.1186/s13033-017-0157-z

**Published:** 2017-08-24

**Authors:** Shameran Slewa-Younan, Anisa Yaser, Maria Gabriela Uribe Guajardo, Haider Mannan, Caroline A. Smith, Jonathan M. Mond

**Affiliations:** 1 Mental Health, School of Medicine, Translational Health Research Institute, Western Sydney University, Locked Bag 1797, Penrith South DC, Sydney, NSW Australia; 2 Centre for Mental Health, Melbourne, School of Population and Global Health, University of Melbourne, Melbourne, Australia; 3 Mental Health, School of Medicine, Western Sydney University, Sydney, Australia; 4 Mental Health, School of Medicine, Translational Health Research Institute, Western Sydney University, Sydney, Australia; 5 Translational Health Research Institute, School of Medicine, Western Sydney University, Sydney, Australia; 6 National Institute of Complementary Medicine, Western Sydney University, Sydney, Australia; 7 Centre for Rural Health, University of Tasmania, Hobart, Australia; 8 School of Medicine, Translational Health Research Institute, Western Sydney University, Sydney, Australia

**Keywords:** Refugees, Afghanistan, Mental health, Trauma, Help-seeking

## Abstract

**Background:**

Psychological trauma, in particular, posttraumatic stress disorder (PTSD) and depression, are highly prevalent among resettled refugees. However, little is known regarding the mental health status and associated help-seeking behaviour of resettled Afghan refugees in Australia.

**Methods:**

A sample of 150 resettled Afghan refugees (74 males; mean age 32.8 years, SD = 12.2) living in Adelaide, South Australia were recruited. Self-reported measures of PTSD, depression, exposure to traumatic events, functional impairment, self-recognition of PTSD symptomatology and help-seeking behaviours were completed. Multivariate analysis of variables associated with help-seeking was conducted.

**Results:**

Forty-four percent of participants met criteria for clinically significant PTSD symptoms and all but one participant reported being exposed to 1 or more traumatic and/or conflict related events, such as ‘losing your property and wealth’. Moreover, 14.7% of participants had symptoms suggestive of clinically significant depression. General practitioners were the most common source of help in relation to mental health problems, with very few participants (4.6%) seeking help from specialist trauma and torture mental health services. Self-recognition of having a PTSD related mental health problem and functional impairment levels were both found to be independent predictors of help-seeking (*p* ≤ .05).

**Conclusions:**

The findings provide further evidence for high rates of PTSD symptomatology and low uptake of mental care among resettled refugees. Poor self-recognition of the presence and/or adverse impact of PTSD symptoms may need to be targeted in mental health promotion programs designed to improve “mental health literacy” and thereby promote early and appropriate help-seeking where this is needed.

## Background

The world is currently experiencing the worst refugee crisis of all times, with record-high numbers of people been forcibly displaced [1]. The United Nations High Commissioner for Refugees (UNHCR) has defined the term *‘refugee’* as ‘a *person who has left their home country as a result of a well*-*founded fear of persecution for reasons of race, religion, nationality, membership of a particular social group or political opinion’* (p. 16) [2]. Refugees are commonly exposed to high levels of violence, trauma, displacement and life disruption due to their unstable situation [2]. Afghanistan and its people have steadily endured multiple conflicts and civil wars since the nineties with many waves of civilians fleeing the country for fear of persecution from the Taliban regime [3]. It is understood that there are over 2,662,954 refugees originating from Afghanistan worldwide, with a further 1,174,306 internally displaced [1]. Australia has been ranked as the third leading resettlement nation worldwide in offering protection to refugees through their Humanitarian Program [4] with Afghanistan being one of the top ten source countries of refugee application to Australia since 2010 [5]. Specifically, there were 12,228 individuals from Afghanistan resettled in Australia, in the period 2010–15 alone [6].

Psychological trauma, namely posttraumatic stress disorder (PTSD) and depression are highly prevalent in individuals of refugee background. In fact, a meta-analysis conducted in multiple refugee groups reported that weighted prevalence of PTSD and depression stood at 30.9 and 30.8%, respectively [7]. Multiple factors are postulated to account for such high prevalence of mental health disorders in refugees. Traditionally, pre-displacement and post-displacement traumas and stressors have been well identified as predictors of poor mental health in such groups [7–10]. However, to date very few studies have reported on the mental health outcomes of Afghan refugees living in Western nations. Of the studies that have been conducted, findings have consistently noted very high rates of mental health disorders ranging from 25.4 to 35% for PTSD and 54.7–57% for depression [11, 12]. This finding of high levels of mental health problems and general psychological distress is further supported in recent mixed methods systematic review of Afghan refugees mental health outcomes [13], that called for continued mental health research with resettled Afghans in order to further understand the relationship between mental health and help-seeking in this group of refugees.

The help-seeking behaviour of refugees experiencing mental health problems also remains under investigated. Help-seeking has been defined as ‘any communication about a problem which is directed toward obtaining support, advice or assistance in times of distress’ (p. 413) [14]. This can comprise of both professional and non-professional help-seeking. The first component is seen as the assistance from professionals who have a legitimate and recognised professional role in providing relevant advice, support and/or treatment, while the second is the assistance from informal social networks, such as friends and family [15]. There is some evidence suggesting that the uptake of mental health care among refugee groups is well below that of individuals with mental health problems in the non-refugee host population [16]. Comparing annual, age-standardised hospital admission rates in Victoria for people born in refugee-source countries and Australian-born people, Correa-Velez and colleagues [16, 17] found that participants from a refugee background were 30% less likely to have mental or behavioural admissions than those born in Australia. An Australian-based study conducted with participants from an Arab background similarly noted low mental health service utilisation [18]. In this study, only 32% of people reporting stress, anxiety and low mood, sought some form of help and an even smaller number, 18% of those with high distress, sought professional help in the previous year [18]. A consistent pattern of low levels of help-seeking was also demonstrated in group of Iraqi refugees attending English tuition classes, where it was noted that only 32.9% of individuals with significant PTSD symptoms sought help [19].

Evidence suggests that poor ‘mental health literacy’ (MHL), the latter defined as ‘knowledge and beliefs about mental health disorders which aid their recognition, management and prevention’ [20], may be a major factor in the low uptake of mental health care among individuals with mental health problems [21]. Findings from recent studies suggest that this includes Iraqi and Afghan refugees being resettled in Australia [22–24]. One aspect of the MHL in refugee samples that appears worthy of further investigation is the ability of these individuals to recognise the signs of a trauma-related problem in themselves. Previous research in community based samples of individuals with symptoms of other mental health problems, such as eating disorders, suggest that self-recognition of mental health problems is poor and that this is a factor in help-seeking behavior, including the low uptake of mental health care [25–28]. To our knowledge, however, self-recognition of PTSD symptomatology, and the potential association of this with help-seeking behavior, has not yet been examined in resettled refugee populations. Findings in this regard may have implications for mental health promotion and/or early intervention programs, for example, the need to target poor MHL among individuals at high risk of or with early signs of PTSD.

With these considerations in mind, the aims of the current study were twofold. First, we sought to report on the mental health status (levels of PTSD and depressive symptomatology and exposure to traumatic events), functional impairment and help-seeking behaviour, of Afghan refugees resettled in Australia. Second, we sought to examine demographic and other variables, including self-recognition of PTSD symptomatology, associated with help-seeking behaviour in this population.

## Methods

### Study design and participants

Participants of this study were Afghan refugee adults resettled in South Australia. Inclusion criteria were having been born in Afghanistan, having left Afghanistan during or after 2000, being fluent in Dari and/or English, and being between the ages of 18 and 70 years. Resettlement during or following 2000 was required in order to establish a more homogeneous sample in terms of exposure to conflict, namely, individuals who were living in Afghanistan following arrival of the Taliban regime. One of the authors (AY) promoted the study among the South Australian Afghan community through networking at Afghan cultural, religious and other Afghan gatherings and by placing flyers (translated into Dari) on the walls of venues (e.g., grocery stores) known to be frequented by the Afghan population in Adelaide (capital of South Australia). The flyer included information concerning the study aims, the time commitment entailed in participation and the inclusion criteria. A combination of convenience and snowball sampling was employed to maximise participation.

Interviews were held between April and September 2013, conducted individually and, most often, in the homes of the participants by author AY (fluent in Dari and English) with each session ranging from 60 to 90 min. Demographic data was also collected as part of the interviews. All participants were provided with (translated) information sheets containing details of local specific mental health services. A food gift voucher in the amount of AUD $25.00 was also provided to all participants upon completion of the survey in appreciation of their time. In addition, a participant information sheet, informing the details of the study, was given. Written consent was obtained from all participants, following a more detailed description of the survey content, prior to commencement of each interview. The study methods were approved by Western Sydney University Human Research Ethics Committee (H10048).

### Measures

#### Assessment of self-recognition and help-seeking

The survey included, in addition to demographic information and measures of mental health and disability levels as described below, a ‘mental health literacy’ (MHL) component in which a vignette was presented of a fictional Afghan refugee ‘Mariam or Ahmad’ (sex of character matched to sex of interviewee), who had been exposed to trauma prior to leaving Afghanistan and who was suffering symptoms of PTSD, followed by a series of questions concerning the problem described. This survey and procedure has been previously reported [23].

Relevant to the purposes of this study were a series of questions embedded in the MHL survey designed to assess self-recognition (both current and past) and help-seeking. For self-recognition, the following question was asked ‘Do you think you might have or ever had a problem such as the one described?’ Participants who responded in the affirmative were deemed to recognise their symptomology. Help-seeking was assessed with a question that asked whether participants had ever sought help for a problem such as the one described. Participant who answered ‘yes’ to this question were asked, from which person(s) or treatment services they had sought this help. A broad range of potential treatment providers was listed for this purpose, including informal sources of help, such as family members and friends and clergy, primary care and allied health practitioners and mental health professionals including refugee-specific outpatient services.

#### Depression symptoms

The Hopkins Symptoms Check List (HSCL-25) was used to measure general anxiety and depression symptoms [29]. The HSCL-25 consists of 25 items in total, 10 items measure anxiety and the remaining 15 items assess for symptoms of depression. Each item is measured in relation to the past week on a Likert scale with choices ranging from 1 (not at all) to 4 (extremely). This scale has been used widely in the studies of war affected displaced refugees including two recent studies of Afghans [29–32]. Only the depression subscale was utilised in this study and participants with a mean score greater than 1.75 were considered to be symptomatic for depressive disorder [29]. Cronbach’s alpha in the current study was .96.

#### Trauma and PTSD symptoms

To assess level of exposure to traumatic events the Afghan War Experience Scale (AWES) was used: This scale asks participants to indicate whether they have experienced each of 17 war-related experiences of violence or loss with answer choices including never (0), once (1), or more that once (2) [33]. Scores on the 17 items are totalled, yielding a possible range of 0–34, with higher total scores reflecting greater exposure to war-related experiences.

PTSD symptomology in relation to the past week was assessed using the 22 item impact of events scale-revised (IES-R). This measure has been used widely to examine psychological trauma [34]. The original version of the scale consists of 22 items with a Likert scale ranging from 0 (not at all) to 4 (extremely). However, in the Afghan adaption of the scale, Miller et al. [33] added a 23rd item to the questionnaire to assess the extent to which participants avoid talking about their symptoms of trauma to avoid upsetting others. However, as this item does not contribute to the final IES-R scores, it was not utilised in this study. The IES-R has demonstrated strong psychometric properties, with the internal consistency reported as .96 [35]. Although not originally designed to be used as a diagnostic assessment tool for PTSD, there have been several studies to date that demonstrated its promise as a brief self-report measure for assessing PTSD [35]. In the largest and most relevant study to date looking at the diagnostic utility of IES-R in two samples of war affected populations (n = 3313 and n = 854), it was noted that a cut- off score ≥34 was able to identify individuals with probable PTSD when compared to the gold standard measure of MINI International Neuropsychiatric Interview (MINI) [36]. Probable cases of PTSD were identified as all those who reported exposure to at least one interpersonal/violent event in AWES and had IES-R score of ≥34. Cronbach’s alpha for IES-R in the current study was .97.

### Disability levels

The World Health Organisation Disability Assessment Scale (WHO DAS-II) [37] was used to examine functional impairment. The WHO DAS-II has been used widely in cross-cultural mental health research and consists of 12 items on a 1 to 5 Likert scale thus generating possible scores of 12–60, with a higher scores indicative of greater functional impairment. The categories are as follows: no disability (0), mild disability (1–4), moderate disability (5–9) and severe disability (10+) [37]. Cronbach’s alpha in the current study was .95.

### Statistical analysis

Analyses were conducted using SAS Version 9.4 [38]. Bivariate analyses examining associations between demographic characteristics such as age, gender, education and marital status and clinical characteristics, namely the number of traumatic events and probable PTSD and depression disorders were undertaken utilising independent samples *t* test, Kruskal–Wallis test, Chi squares or correlations as appropriate. Collinearity between PTSD and depression was examined using the nonparametric Wilcoxon sum rank test and found not significant. Since help-seeking is likely to be multifactorial, a multiple logistic regression using the variables reported in Table 3 was undertaken using penalised likelihood as reported by Firth [39] to account for sample size. The statistical significance of each covariate was based on profile likelihood confidence interval rather than Wald p-value or Wald confidence interval as recommended by Allison [40]. The significance level of each covariate was set to 5% and McFadden’s pseudo R^2^ [41, 42] was utilised instead of Hosmer–Lemeshow statistic due to sample size limitations [43]. There was no missing data for the predictors used in multiple logistic regression analysis.

## Results

Interviews were conducted with a total of 150 participants over 7-month data collection period. Their demographic characteristics are shown in Table 1.

**Table 1:** Demographic characteristics of participants (n = 150)

Characteristics	Afghan refugees N (Total = 150)	%
Gender		
Male	74	49.3
Female	76	50.7
Age in years, mean (SD)	32.8 (12.3)	–
Years of education, mean (SD)	6.1 (5.2)	–
Months in Australia, mean (SD)	71.4 (49.6)	–
Months externally displaced, mean (SD)	67.5 (68.8)	–
Arrival status to Australia		
Refugee	64	42.7
Asylum seeker	52	34.7
Immigrant	34	22.7
Marital status		
Never married	38	25.3
Married/partner	97	60.6
Divorced	2	1.3
Widowed	13	8.6

### Total potentially traumatic events (PTE’s) experienced

All but one individual reported being exposed to at least one PTE prior to leaving Afghanistan, with 125 participants reporting exposure to 10 or more PTEs. The most commonly reported experiences were being forced to flee your home (99.3%), losing your property and wealth (96.6%), loss of family income (95.3%) and disappearance of family member (90.7%). Total PTEs varied as a function of gender with more males (median of 15 PTE) experiencing greater numbers of traumatic events than females (median of 13 PTE); t (148) = 3.34 p = .001. Similarly, older age and lower levels of education were significantly associated with exposure to more traumatic events, *r*_*t*_ (148) = .22, p < .001. and *r*_*t*_ (148) = −.28, p = .000 respectively. Finally, the number of traumatic events experienced differed significantly across marital status [H(4) = 11.53, p = .021]. The post hoc analysis showed that those married were exposed to more traumatic events (median of 14 PTE) compared to those never married (median of 13 PTE) (p = .012).

### PTSD and depression symptoms

Fourty-four percent of participants met the threshold (cut off score >34 and exposure to interpersonal/violent event) for clinical significant symptoms of PTSD according to the IES-R. Referring to the cut-off score >1.75 of the depression subscale of HSCL-25, 14.7% had symptoms suggestive of probable depression. There were no statistical significant associations between PTSD status and demographic characteristics. However, those with probable depression had significantly higher levels of education (median of 10 years) than those without probable depression (median of 5.5 years); t (148) = −2.27, p = .025.

### Help-seeking behaviour

Table 2 details sources of help-seeking for the sample. When the whole of sample participants were asked whether they had ever sought help for a problem such as one described in the vignette, almost half the sample (49.3%) reported in the affirmative. Of these, the top three sources for seeking help were the ‘General Practitioner’ (53.3%) followed by ‘Psychologist’ (38.6%) and ‘Psychiatrist’ (33.3%). It is interesting to note that only 4.6% of sample reported seeking help from specialist trauma and torture mental health services. When help-seeking was considered in those participants that had clinically significant PTSD symptoms, the rates of help-seeking were similar, with 50% reporting having ever sought help for a mental health problem. Further, within this sub sample, assistance was sought from the same health professionals albeit at a lower rate, with 46.7% seeking assistance from a ‘General Practitioner’ followed by 31.7% consulting a ‘Psychiatrist’ and a similar 31.7% seeing a ‘Psychologist’. Approximately 10% of the sample with probable PTSD reported having been treated by specialist trauma and torture mental health services.

**Table 2:** Clinical characteristics and help-seeking sources

Clinical characteristics	N	%
Probable PTSD (IES-R^a^)	60	44.1
Probable depression (depression subscale of HSCL-25)	22	14.7
Exposure to traumatic events (AWES; M and SD of PTEs)	12.9	3.3
Level of disability (WHO –DAS II)	–	–
No disability	28	18.7
Mild disability	19	12.7
Moderate disability	33	22
Severe disability	70	46.7
Self-recognition of mental health problem		
No	62	41.3
Yes	88	58.7
Sources of help^b^		
General Practitioner	80	53.3
Psychologist	58	38.6
Psychiatrist	50	33.3
Family member	14	9.3
Community mental health service	14	9.3
STTARS^c^	7	4.6
Close male friend	6	4
Close female friend	3	2
Religious leader	3	2
Telephone counselling line	2	1.3
Afghan social group	1	.6

^a^ *n* = 136

^b^Total percentage is greater than 100% due to the fact that participants were able to select more than one source of help

^c^Survivors of Torture and Trauma Assistance and Rehabilitation Service, South Australia

### Multivariate analysis of variables associated with help-seeking

Table 3 presents the results of logistic regression analysis examining predictors associated with help-seeking.

**Table 3:** Odds ratios and 95% profile confidence intervals of help-seeking in Afghan refugees

Predictors	Odds ratio	95% CI
Age	.980	.935–1.026
Gender		
Female	1.00	
Male	1.437	.629–3.345
Years of education	1.047	.954–1.154
Self-recognition of mental health problem^a^		
No	1.00	
Yes	4.857	12.160–11.507
Probable PTSD		
No	1.00	
Yes	1.059	.448–2.518
Probable depression		
No	1.00	
Yes	1.462	.412–5.425
Exposure to traumatic events	1.016	.852–1.217
Disability score (WHO-DAS II)^a^	1.088	1.026–1.161

^a^Indicates significant at α = .05

The results indicated that only two predictors, self-recognition and functional disability scores, were statistically significant in determining help-seeking. Resettled Afghan refugees who identified as having a problem such as one described in vignette were 4.9 times more likely to seek help than those who did not. Further, it found that for every one unit increase in disability score it was associated with an 8.8% increase in the odds of seeking help. The results based on pseudo R^2^ show that the logistic regression model explains 22% of the variability of our sample’s help-seeking behaviour.

## Discussion

A call for research seeking to examine the mental health and help-seeking behaviour of Afghan refugees was noted in a systematic review of Afghan mental health literature [13], thus highlighting the potential significance of this current study. Our findings indicated that almost every participant reported being exposed to one or more potentially traumatic events and approximately 44% of the group reported symptoms consistent with a diagnosis of PTSD and 15% having probable depression. Preferred sources of help-seeking were found to be “general practitioners”, ‘psychiatrist’ and ‘psychologists’. Only two factors were found to significantly predict help-seeking, self-recognition and increased levels of functional impairment.

Of the few studies that have reported on the mental health status of resettled Afghan refugees in Western nations, noted rates of PTSD and depression are very high, which generally consistent with our findings. Although not directly comparable, we can cautiously refer to the reported rates of PTSD and depression in the general Australian population to further contextualise our results. In the 2007 National Survey of Mental Health and Wellbeing (NSMHW) it was noted that 12 months rate for PTSD was 6.4% [44]. Thus our 44% rate of probable PTSD would indicate that Afghan refugees are over six times more likely to have PTSD. Further, this rate of probable PTSD in our Afghan sample was not found to be associated with any specific demographic characteristics, indicating it’s wide prevalence in this community. In light of the rates of exposure to PTEs reported in our sample (mean of 12 PTEs), such significant levels of PTSD symptomology is concerning but not surprising. However, our finding that 14.7% of our sample had probable depression, a figure lower than previously reported rates of as high as 57%, is unexpected and warrants further discussion. Referring to the NSMHWB data, 12 months prevalence rate of depression was found to be 6.3% in the Australian public [44]. This indicates that our sample was at least twice as likely to have depression as the general Australian public. As such health providers and other key stakeholders should be mindful of possibility that a disproportionate number of resettling Afghan refugees may present with clinically significant mental health problems. Another factor of note is our finding that those with probable depression had significantly higher levels of education than those without. Previous studies of refugees have demonstrated resettlement challenges such as difficulty with recognition of qualifications and loss of status [7, 45] are associated with higher depression levels thus providing a possible explanation for our finding.

A second aim of the current research was to elucidate the help-seeking behaviour of resettled Afghan refugees. Our findings indicated that almost half our sample had sought help for a problem similar to that described in the vignette with professional health providers being the top sources of preferred help. When only the sample with probable PTSD was considered, help-seeking rates were similar (50%) and the top preferred sources of help remained consistent. While help-seeking in our Afghan sub sample with probable PTSD is higher at 50% compared with rates found in our previous study of Iraqi refugees where 32.9% of those with probable PTSD reported having sought help [19], it is still low and remains far below the ideal with all who demonstrate clinically significant symptoms seeking help. Of importance is our finding of the low levels of reported uptake of specialist trauma and torture mental health services, which ranged from 4.6% from the whole sample to 10% in the sub sample with probable PTSD. Given that these services have been founded with the mission of providing treatment and rehabilitation for torture and trauma affected refugees, such low levels of uptake are concerning and may reflect poor awareness of the services amongst target populations.

In multivariate analysis, only self-recognition of a probable mental health problem and levels of functional impairment were independently associated with help-seeking. While the finding that levels of functional impairment were independently associated with help-seeking is not surprising, it is useful to confirm that resettled refugees with mental health problems are similar in this regard to individuals with mental health problems more generally [46]. Further, there is now good evidence from community-based research that early, appropriate help-seeking is association with improved mental health outcomes [21]. The current findings support the need for health promotion and/or early intervention programs designed to improve the MHL of resettled refugees relating to PTSD symptoms and thereby increase the likelihood that help is received when needed. Further, our findings suggest that individuals’ ability to recognise that they may have a mental health problem may be one specific aspect of “poor MHL” worthy of addressing in such programs. Ideally, programs designed to improve MHL among resettled refugees would be integrated with efforts to improve the MHL of individuals assisting in the resettlement process (e.g., [47]) for maximum impact.

Strengths of this study include the administration of the survey instruments via in-person interviews and the recruitment of participants by a researcher who could speak the language, and was of the same nationality as participants, all of which helped to establish rapport and trust and thereby improved the recruitment process and the quality of the data collected. This may have also contributed to recruitment of a fairly equal proportion of male and female participants, another strength of the paper. This is also the first paper to our knowledge that seeks to report on help-seeking behavior in resettled Afghan refugees and variables associated with this. A number of limitations should also be noted. Firstly, self-report measures of PTSD and depression symptomology were utilised rather than more precise standardised clinical diagnostic interviews, primarily due to limitations of sampling instruments available in Dari. Secondly, study participants were volunteers rather than individuals recruited by means of random sampling. This may limit the generalisability of the findings, although the lack of a clear sampling frame and limitations with Census data is problematic in research of this kind [48]. Finally, self-recognition of a probable mental health problem was assessed with a single item responses to which might depend, in part, on the specific wording of both this item and that employed in the vignette [25–27]. However, the finding that self-recognition was independently associated with help-seeking is consistent with findings from community-based studies of individuals with other mental health problems [25–27].

## Conclusions

Despite the high levels of trauma-related mental health problems, it appears that the uptake of mental health care is problematic among Afghan refugees resettled in Australia. Poor self-recognition of the presence and/or adverse impact of PTSD symptoms among sufferers may be one aspect of MHL worthy of targeting in health promotion and/or early intervention programs designed to promote early, appropriate help-seeking where this is needed.

## Abbreviations

PTE: potentially traumatic events
PTSD: posttraumatic stress disorder
MHL: mental health literacy
AWES: Afghan War Experience Scale
IES-R: Impact of Events Scale-Revised
HSCL–25: Hopkins Symptoms Checklist–25
NSMHWB: Australian National Survey of Mental Health and Wellbeing
