# Erratum to: The mental health and help-seeking behaviour of resettled Afghan refugees in Australia

**DOI:** 10.1186/s13033-017-0163-1

**Published:** 2017-09-21

**Authors:** Shameran Slewa-Younan, Anisa Yaser, Maria Gabriela Uribe Guajardo, Haider Mannan, Caroline A. Smith, Jonathan M. Mond

**Affiliations:** 1 Mental Health, School of Medicine, Translational Health Research Institute, Western Sydney University, Locked Bag 1797, Penrith South DC, Sydney, NSW Australia; 2 Centre for Mental Health, Melbourne, School of Population and Global Health, University of Melbourne, Melbourne, Australia; 3 Mental Health, School of Medicine, Western Sydney University, Sydney, Australia; 4 Mental Health, School of Medicine, Translational Health Research Institute, Western Sydney University, Sydney, Australia; 5 Translational Health Research Institute, School of Medicine, Western Sydney University, Sydney, Australia; 6 National Institute of Complementary Medicine, Western Sydney University, Sydney, Australia; 7 Centre for Rural Health, University of Tasmania, Hobart, Australia; 8 School of Medicine, Translational Health Research Institute, Western Sydney University, Sydney, Australia

## Erratum to: Int J Ment Health Syst (2017) 11:49 DOI 10.1186/s13033-017-0157-z

Following publication of the original article [1], the authors reported that there was a mistake in a figure included in Table 3, which was not in the submitted version. It should read 2.160–11.507—not 12.160–11.507.

The original article has been corrected.
